# Radiation to supraclavicular and internal mammary lymph nodes in breast cancer increases the risk of stroke

**DOI:** 10.1038/sj.bjc.6604902

**Published:** 2009-03-03

**Authors:** G Nilsson, L Holmberg, H Garmo, A Terent, C Blomqvist

**Affiliations:** 1 Department of Oncology, Radiology and Clinical Immunology, Section of Oncology, University Hospital, SE-751 85 Uppsala, Sweden; 2 Regional Oncologic Centre and Department of Surgery, University Hospital, SE-751 85 Uppsala, Sweden; 3 Division of Cancer Studies, King's College London, School of Medicine, London, UK; 4 Department of Medical sciences, University Hospital, SE-751 85 Uppsala, Sweden

**Keywords:** breast cancer, stroke, cerebral infarction, radiotherapy, cerebrovascular

## Abstract

The aim of this study was to assess whether adjuvant treatment of breast cancer (BC) affects the risk of stroke, and to explore radiation targets and fraction doses regarding risk and location of stroke. In a Swedish BC cohort diagnosed during 1970–2003, we carried out a nested case–control study of stroke after BC, with relevant details extracted from medical records. The odds ratio (OR) for radiotherapy (RT) *vs* that of no RT did not differ between cases and controls (OR=0.85; confidence interval, CI=0.6–1.3). Radiotherapy to internal mammary chain (IMC) and supraclavicular (SCL) lymph nodes *vs* that of no RT was associated with a higher, although not statistically significant, risk of stroke (OR=1.3; CI=0.8–2.2). In a pooled analysis, RT to IMC and SCL *vs* the pooled group of no RT and RT to breast/chest wall/axilla (but not IMC and SCL), showed a significant increase of stroke (OR=1.8; CI=1.1–2.8). There were no associations between cancer laterality, targets of RT, and location of stroke. The radiation targets, IMC and SCL, showed a statistically significant trend for an increased risk of stroke with daily fraction dose. Our finding of a target-specific increased risk of stroke and a dose-response relationship for daily fraction dose, indicate that there may be a causal link between RT to the IMC and SCL and risk of stroke.

Randomised clinical trials (Overgaard *et al*, 1997, 1999; Ragaz *et al*, 1997) and meta-analyses by the Early Breast Cancer Trialists' Collaborative Group (Clarke *et al*, 2005) have shown a benefit of post-operative radiotherapy (RT) in breast cancer (BC), by reducing local recurrences and BC deaths. On the other hand, there seems to be an excess of vascular deaths in women who were given RT (Clarke *et al*, 2005). Radiotherapy to the supraclavicular (SCL) lymph nodes includes the proximal part of the carotid artery in the photon field. Studies of head and neck cancer have shown an increased risk of carotid stenosis, followed by ischaemic stroke after RT (Cheng *et al*, 2000; Dorresteijn *et al*, 2002; Haynes *et al*, 2002).

Our earlier study (Nilsson *et al*, 2005) showed a statistically significant increase of 12% of stroke in a large BC cohort. Another study (Jagsi *et al*, 2006) found similar results, whereas two others did not (Hooning *et al*, 2006; Woodward *et al*, 2006). None of the last three studies showed a statistically significant increase of stroke among women given RT to the SCL.

In our case–control study, we aimed to assess whether any adjuvant treatment of BC, such as RT, tamoxifen, and chemotherapy, affects the risk of stroke, and to explore radiation targets and radiation fraction doses regarding risk and location of stroke.

## Patients and methods

We conducted a case–control study of the first stroke after BC nested in a cohort of women with BC. The cohort comprises 4689 women with a first invasive BC diagnosed during 1970–2003 residing in Uppsala County, Sweden, at diagnosis and registered in the Swedish Cancer Register, which has 98% coverage (Mattsson and Wallgren, 1984). To identify stroke after BC, the cohort was linked, by Personal Registration Numbers, to the Swedish Hospital Discharge Register, containing all in-patient health care events. The definition of stroke was based on the International Classification of Disease (ICD) codes (Table 1). The ICD codes were grouped into the following subtypes: ischaemic stroke, cerebral haemorrhage, and ill-defined cerebrovascular lesion (Lawlor *et al*, 2002).

We found 316 eligible women with an invasive BC followed by hospitalisation for a stroke during the period 1970–2003, of whom 34 were excluded after record review, leaving 282 cases for analysis. The exclusion criteria were that a stroke could not be verified in the medical records, or that the medical records revealed a history of stroke also before the BC diagnosis. A total of 12 women were excluded, as they did not have an invasive BC, or as their BC was diagnosed before 1970.

For each case, one control was chosen by incidence density sampling (Rothman, 1998) ensuring that the probability of being chosen is proportional to the time at risk in the cohort. The control was sampled at random, among those who were alive and without a history of hospitalisation for a stroke at the date of the stroke, for the case. We refer to this date as index date for the controls. Cases were eligible as control subjects before their stroke event. We found a stroke diagnosis in two primarily selected controls before the index date. These patients were not in the risk set and, consequently, were not eligible as controls. In addition, 11 controls had no BC, or had BC diagnosed before 1970. For the 13 women, new controls were selected for each respective case by the same statistical method, leaving a total of 282 controls.

From the medical records we obtained information on BC, according to the International Union Against Cancer (UICC) TNM classification (6th edn) (tumour size, node status, presence of distant metastases, and laterality), and details of treatments.

With regard to RT, we collected information from the chart about target areas: remaining breast tissues after breast conserving surgery (BCS), chest wall after mastectomy, lymph nodes in the axilla, internal mammary chain (IMC), and SCL area. Data regarding daily fraction radiation doses and total doses were also collected. In respect of exposure to radiation, the subjects were separated into three groups: (1) no RT; (2) RT to breast/chest wall/axilla, but not to IMC/SCL; and (3) RT to IMC/SCL, irrespective of RT to other targets. In the following, we refer to these groups as: (1) ‘No RT’; (2) ‘RT except IMC/SCL’; and (3) ‘RT to IMC/SCL’.

During the study period 1970–2003, several different RT regimens have been used. The thoracic wall has been treated with low energy electrons during the whole period. The fraction schemes were: 3 Gy × 15=45 Gy (1970–1985), 2.3 Gy × 20=46 Gy (1986–1996), and thereafter 2 Gy × 25=50 Gy. Breast conserving surgery was started in the year of 1982 in our health care region. The remaining breast tissues were then treated with two opposed tangential photon fields 2 Gy × 27=54 Gy. From 1997 and further, the fractionation scheme was 2 Gy × 25=50 Gy.

The lymph nodes have been treated with different techniques and fractionation during this period. In the period 1970–1972, small–sized frontal Cobolt-60 photon fields to cover the SCL and IMC were given. One such small field of 7 Gy was given each day, and in consecutive days a chain of fields were given to cover the targets. The axilla was treated with photons (Cobolt-60), 4 Gy × 7 in a frontal field and 4 Gy × 6 in a dorsal field. In the period 1973–1976, IMC, SCL, and axilla were treated with a frontal Cobolt-60 photon field of 4 Gy × 10=40 Gy. The IMCs were treated simultaneously using a frontal field of electrons 3 Gy × 5=15 Gy, whereas the axilla was given 4 Gy × 4–5=20–24 Gy in a dorsal photon field.

In the period 1977–1985, IMC, SCL, and axilla were treated using a frontal Cobolt-60 photon field of 3.5 Gy × 9=31.5 Gy. The IMC and SCL were treated simultaneously using a frontal field of electrons 3 Gy × 5=15 Gy, whereas the axilla was given 4 Gy × 6=24 Gy in a dorsal photon field. In the 1986–1994 period, IMC, SCL, and axilla were treated using a frontal photon field of 2.5 Gy × 12=30 Gy. The IMC and SCL were treated simultaneously using a frontal field of electrons 2.5 Gy × 8=20 Gy, whereas the axilla was given 3.2 Gy × 8=25.6 Gy in a dorsal photon field.

In 1995 and after, treatment of the lymph nodes was dose planned, and all the included nodes were given 2 Gy × 27=54 Gy. In 1997 and later, the nodes were given 2 Gy × 25=50 Gy.

Information regarding adjuvant endocrine treatment (specified as tamoxifen or aromatase inhibitor), chemotherapy data (type of regimens and number of courses), and recurrences (local or distant) were abstracted from the medical records. For our stroke classification, we used all available information in the medical records, namely, medical history including clinical presentation, results of computed tomography (CT) scan and MRI, angiography, surgery reports, and autopsy records. We reviewed the records to decide whether the stroke was in the vertebrobasilar or carotid territories (Bamford *et al*, 1991). For carotid strokes, we also registered left or right hemisphere location. In the 32 cases, in which the classification and location was not obvious, two independent reviewers carried out a consensus evaluation, but this was still not possible in five of the cases. We registered the presence of atrial fibrillation, documented by electrocardiography, at the time of stroke among the cases.

Odds ratios (ORs) with 95% confidence intervals (CIs), in the 1 : 1 matched case–control study, were calculated using conditional logistic regression (Breslow and Day, 1980), adjusting for age in four categories (<60, 60–69, 70–79, and 80+ years) at BC diagnosis; this is done using Cox's regression (Cox, 1972). Tests of independence between laterality of BC and stroke for various RT regimens were carried out using Fisher's exact test. All statistical analyses were carried out using the statistical program package R (Ihaka, 1996).

## Results

The characteristics of cases and controls are described in Table 2. Cases were significantly older. The mean age at BC diagnosis was 71.5 years (s.d., 11.0 years) for cases and 58.7 years (s.d., 13.2 years) for controls. The median follow-up period was 6.2 years (25th percentile 2.8 years; 75th percentile 11.0 years) for cases and 5.5 years (25th percentile 1.9 years; 75th percentile 9.3 years) for controls.

The cases had slightly more advanced cancers than the controls. Stage I cancer was more common among the controls. Only 1% of all subjects had distant metastasis at BC diagnosis with no difference between cases and controls. Less than 10% of the cases and controls had distant metastasis at the time of stroke or at the index date (controls). Furthermore, <5% of subjects had local recurrences at the time of stroke or at the index date. Cases and controls did not differ regarding incidence of local or distant recurrences.

Only four cases developed other cancers before their stroke, whereas controls did not. The cancers were non-Hodgkin lymphoma, ovarian cancer, small intestinal carcinoid tumour, and gastric cancer, respectively.

Almost 80% of the strokes originated in the carotid arteries and 20% in the vertebrobasilar system (Table 3). Computed tomography of the brain was carried out in 73% of the cases. Of these cases, 62% had ischaemic stroke, of which 48% had cerebral infarction and 14% had transient cerebral ischaemia. Ten percent of the cases had haemorrhagic stroke (2%, subarachnoidal haemorrhage and 8%, intracerebral haemorrhage). Of these cases, 28% had ill-defined stroke, mostly diagnosed in ‘the pre computer tomography era’, and 22% of the cases had atrial fibrillation at the time of their stroke.

We evaluated the relationship between stroke after BC and adjuvant treatments (Table 4). In the first analysis, not adjusted for age, we found no association between any BC treatment and stroke (data not shown). As the cases were significantly older than the controls, we adjusted the ORs for age. The overall OR for RT did not differ significantly from unity. However, as the analyses were stratified on RT type, differences emerged. Radiotherapy, except IMC/SCL compared with that of no RT was associated with a lower risk of stroke. Moreover, RT to IMC/SCL *vs* that of no RT was associated with a higher, although not significant, risk of stroke (OR=1.3; CI=0.8–2.2). When pooling the groups, no RT and RT, except IMC/SCL, a *post hoc* comparison of RT to IMC/SCL *vs* the pooled group showed a significant increase of stroke with an age-adjusted OR=1.8 and CI=1.1–2.8.

We also repeated the analyses stratifying on cerebral haemorrhage in one group (*n*=29) and ischaemic stroke plus ill-defined cerebrovascular lesion in another group (*n*=253); the latter showing a same pattern as for all types of stroke, and significantly increased risk of stroke among the subjects receiving RT to IMC/SCL *vs* that of no RT and RT, except IMC and SCL (OR=1.9; CI=1.2–3.2). No significant association between RT and haemorrhagic stroke was seen, although the CIs were wide.

Adjuvant tamoxifen was used by few women during the study period, that is ∼15% of the subjects, and the associated OR for stroke was as for chemotherapy below unity, but not in a statistically significant way.

Tamoxifen was used for either 2 or 5 years. Among the cases, 13/46 had tamoxifen for 2 years, whereas 33/46 had tamoxifen for 5 years. For the controls, the numbers were 10/43 and 33/43, respectively. Only one woman received an adjuvant aromatase inhibitor.

When investigating laterality, the analysis was restricted to cases with right or left carotid strokes, but no significant association was found between the BC laterality, targets of RT, and the location of the stroke (Table 5).

We further investigated dosage effects of RT, measured by the daily fraction dose, to different targets and risk of stroke (Table 6). The RT dosage administered to the remaining breast, after breast conservation, was standardised with very similar doses for all who were treated, thus a dose-response relation could not be evaluated (data not shown). However, for IMC and SCL, there was a significant trend for higher risk of stroke with increasing daily fraction dose, especially marked for high doses (⩾4 Gy).

## Discussion

The main finding in our study is a statistically significant (OR=1.9) increase of stroke among women with BC, who had received RT to IMC and SCL. The risk was dose dependent and pertained to ischaemic and ill-defined stroke, but not to haemorrhagic stroke (although, we caution that the statistical precision for haemorrhagic stroke is low). There was no increased risk seen for the RT group as a whole or after exposure to hormonal treatment or chemotherapy. However, in the studied time period, few women were recommended for systematic adjuvant treatments.

This study is based on population-based registries with high coverage (Mattsson and Wallgren, 1984). This study covers a long period of follow-up. The medical records could be retrieved for virtually all cases and controls. We have no reason to believe that cases and controls were followed differently with respect to cerebral vascular disease.

The selection of controls followed standard incidence density-based sampling nested within a well-defined cohort (Rothman, 1998). We deliberately avoided matching for age, stage of disease, and time period of diagnosis, as indications and methods for RT are associated with these factors. Indeed, our findings indicate that such a matching would have entailed over-matching, and the risk of stroke may have been underestimated. Thus, we aimed to fulfil one of the basic methodological rules in a case–control study: the controls will be selected against criteria, which are not associated with a likelihood of or type of exposure.

Of all the strokes, ∼90% are ischaemic and 10% are haemorrhagic (Rothwell *et al*, 2005). This should also be true regarding ill-defined stroke. Thus, we deemed it appropriate to analyse ischaemic and ill-defined stroke together.

Of the cases, 22% had atrial fibrillation at the time of their stroke, corresponding well to epidemiological studies (Sandercock *et al*, 1992). We have no information on cardiovascular risk factors, such as hypertension, smoking, and hyperlipidaemia at the time of RT. However, if anything, it is likely that these factors are associated with more severe co-morbidity and, thus, may be associated with a contraindication for RT, thereby driving our estimates to the conservative side. For example, RT, which was not delivered to IMC and SCL, was associated with a lower risk of stroke that probably reflects a selection bias. There is no plausible biological explanation for RT to decrease the risk of stroke. Most women in this group underwent RT to the remaining breast after breast conservation; the reasons for avoiding RT after breast conservation, during this period, include a low-performance status and serious cardiovascular disease.

Given the possibility of selection bias mentioned above, we deem it likely that RT not delivered to IMC and SCL is associated with either low or no risk of stroke. Furthermore, we found no association between BC laterality and the corresponding hemisphere location of the carotid stroke. Thus, if there is a direct causal link between RT and stroke, we speculate that it is the dose to the superior part of the heart and the aortic arch (corresponding to the IMC radiation field) that is detrimental rather than the dose to the proximal part of the carotid artery (SCL radiation field). In that way, IMC radiation would increase the risk of atherosclerosis (Schultz-Hector and Trott, 2007) and, furthermore, thrombo-embolic stroke and embolus may follow the blood stream to either the right or left carotid artery, irrespective of BC laterality and SCL irradiation. However, we could not analyse the targets, IMC or SCL, as separate risk factors, as most subjects received RT to both targets combined. The distribution of stroke location is similar to that in epidemiological studies (Bamford *et al*, 1991), neither supporting, nor making a case against a causal explanation for RT as a risk factor for stroke.

In contrast to our findings, another study found a significant association between tamoxifen and stroke, but not between RT to SCL and stroke (Hooning *et al*, 2006). However, the subjects were ∼20 years younger, and the study group was restricted to 10-year BC survivors. In two studies (Jagsi *et al*, 2006; Woodward *et al*, 2006), there was no association between RT to SCL and stroke, but no information regarding RT to IMC was given. A recent study (Woodward *et al*, 2008) found no increase of carotid stenosis after radiation, measured by ultrasound.

Our result agrees with another study (Bowers *et al*, 2005), which showed a large increase of stroke, Relative Risk=4.3, among childhood Hodgkin survivors who had received mantle irradiation, involving IMC and SCL bilaterally. In keeping with our speculation that IMC radiation is detrimental, 50% of the Hodgkin survivors with stroke had concomitant heart or valve problems, predisposing to cardio-embolic stroke.

High daily fraction radiation doses increase the risk of late toxicity, such as myocardial infarction (Cuzick *et al*, 1994), lymph oedema, and brachial plexopathy (Johansson *et al*, 2002). Similarly, we found a dose-dependent relationship between radiation dose to IMC and SCL and stroke (Table 6). It is difficult to compare old RT techniques with modern techniques, as generally hypo fraction was used in the former. When using the older techniques no dose planning was used, implicating an increased risk of radiation exposure to other organs in the mediastinum, namely, the heart and the aortic arch and its branches. Furthermore, the radiation volumes were larger and the dose homogeneity was poorer.

Taken together, our earlier finding of a 12% increased risk of stroke in relative terms after BC diagnosis (Nilsson *et al*, 2005), the present target-specific increased risk of a stroke, a dose-response relationship for daily fraction dose, and a suggestion of an increased risk of ischaemic stroke, indicate that there may be a causal relationship between RT to the IMC and SCL and risk of stroke. Together with other studies, our findings indicate that an increased risk of stroke has to be taken into consideration in view of giving RT to IMC or SCL. The ongoing randomised European Organisation for Research and Treatment of Cancer (EORTC) study regarding RT to IMC will provide important data regarding that risk; however, these effects will take a long time to appear.

## Figures and Tables

**Table 1:** ICD codes and definition of subtypes of stroke

**ICD 8**	**(1970–1986)**	**ICD 9**	**(1987–1996)**	**ICD 10**	**(1997–2000)**
*Ischaemic stroke*					
432	Occlusion of pre-cerebral arteries	433	Occlusion and stenosis of pre-cerebral arteries	G45	Transient cerebral ischaemic attacks and related syndromes
433	Cerebral thrombosis	434	Occlusion of cerebral arteries	G46	Vascular syndromes of brain in cerebrovascular diseases
434	Cerebral embolism	435	Transient cerebral ischaemia	I63	Cerebral infarction
435	Transient cerebral ischaemia				
437	Generalised ischaemic cerebrovascular disease				
					
*Cerebral haemorrhage*					
430	Subarachnoid haemorrhage	430	Subarachnoid haemorrhage	I60	Subarachnoid haemorrhage
431	Cerebral haemorrhage	431	Intracerebral haemorrhage	I61	Intracerebral haemorrhage
		432	Other and unspecified intracranial haemorrhage	I62	Other non-traumatic intracranial haemorrhage
					
*Ill-defined cerebrovascular lesion*					
344	Other cerebral paralysis	344	Other paralytic syndromes	I64	Stroke, not specified as haemorrhage or infarction
436	Acute but ill-defined cerebrovascular disease	436	Acute but ill-defined cerebrovascular disease	I65	Occlusion and stenosis of pre-cerebral arteries, not resulting in cerebral infarction
438	Other and ill-defined cerebrovascular disease	437	Other and ill-defined cerebrovascular disease	I66	Occlusion and stenosis of cerebral arteries, not resulting in cerebral infarction
		438	Late effects of cerebrovascular disease	I67	Other cerebrovascular diseases
				I69	Sequelae of cerebrovascular disease

ICD=International Classification of Disease.

**Table 2:** Subjects characteristics

	**Cases (*n*=282)**	**Controls (*n*=282)**
*Year of BC diagnosis*		
1970–1974	42 (14.9)	34 (12.1)
1975–1979	47 (16.7)	39 (13.8)
1980–1984	53 (18.8)	49 (17.4)
1985–1989	56 (19.9)	56 (19.9)
1990–1994	47 (16.7)	50 (17.7)
1995–2003	37 (13.1)	54 (19.1)
		
*Years of follow-up*		
<2	50 (17.7)	78 (27.7)
2–5	67 (23.8)	56 (19.9)
5–10	81 (28.7)	87 (30.9)
10–20	61 (21.6)	54 (19.1)
20+	23 (8.2)	7 (2.5)
		
*Age at BC diagnosis (years)*		
<60	40 (14.2)	148 (52.5)
60–69	65 (23.0)	68 (24.1)
70–79	113 (40.1)	44 (15.6)
80+	64 (22.7)	22 (7.8)
		
*T-stage*		
T1	143 (50.7)	163 (57.8)
T2	79 (28.0)	59 (20.9)
T3–4	13 (4.6)	21 (7.4)
Tx	47 (16.7)	39 (13.8)
		
*N-stage*		
N0	167 (59.2)	200 (70.9)
N+	71 (25.2)	59 (20.9)
Nx	44 (15.6)	23 (8.2)
		
*M-stage*		
M0	280 (99.3)	280 (99.3)
M1	2 (0.7)	2 (0.7)
		
*Stage*		
I	94 (33.3)	128 (45.4)
II	92 (32.6)	82 (29.1)
III	14 (5.0)	12 (4.3)
IV	2 (0.7)	2 (0.7)
Missing	80 (28.4)	58 (20.6)
		
*BC-side*		
Right	148 (52.5)	147 (52.1)
Left	134 (47.5)	135 (47.9)
		
*Distant metastasis before stroke*		
No	259 (91.8)	266 (94.3)
Yes	23 (8.2)	16 (5.7)
		
*Local recurrence before stroke*		
No LR	272 (96.5)	269 (95.4)
LR	10 (3.5)	13 (4.6)

BC=breast cancer; LR=local recurrence.

**Table 3:** Case characteristics

	**Cases (*n*=282)**
*Stroke diagnosis*	
Ischaemic stroke	174 (61.7)
*Transient cerebral ischaemia*	*40 (14.2)*
*Cerebral infarction*	*134 (47.5)*
Cerebral haemorrhage	29 (10.3)
*Subarachnoid haemorrhage*	*7 (2.5)*
*Intracerebral haemorrhage*	*22 (7.8)*
Ill-defined cerebrovascular lesion	79 (28.0)
	
*Location of stroke*	
Carotid right hemisphere	105 (37.2)
Carotid left hemisphere	115 (40.8)
Carotid undefined hemisphere	5 (1.8)
Vertebrobasilar	52 (18.4)
Not defined	1 (0.4)
Insufficient medical records	4 (1.4)
	
*Mode of verification*	
Computed tomography	206 (73.0)
Autopsy without computed tomography	7 (2.5)
Medical records	65 (23.0)
Insufficient medical records	4 (1.4)
	
*Atrial fibrillation*	
Yes	63 (22.3)
No	215 (76.2)
Insufficient medical records	4 (1.4)

**Table 4:** ORs and 95% CIs for stroke and subtypes of stroke associated with adjuvant therapy

	**Cases**		**Controls**		**Stroke, age adjusted**		**Cerebral haemorrhage, age adjusted**		**Ischaemic stroke and ill-defined cerebrovascular lesion, age adjusted**	
	***N***	**(%)**	***N***	**(%)**	**OR**	**95% CI**	**OR**	**95% CI**	**OR**	**95% CI**
*RT*										
No	125	(44.3)	91	(32.3)	ref		ref		ref	
Yes	157	(55.7)	191	(67.7)	0.85	(0.56, 1.30)	1.39	(0.38, 5.07)	0.79	(0.50, 1.24)
										
*RT*										
No RT	127	(45.0)	92	(32.6)	ref		ref		ref	
RT, except IMC/SCL	58	(20.6)	97	(34.4)	0.45	(0.25, 0.79)	1.52	(0.35, 6.59)	0.34	(0.18, 0.65)
RT to IMC/SCL	97	(34.4)	93	(33.0)	1.32	(0.80, 2.19)	1.23	(0.25, 5.96)	1.33	(0.77, 2.28)
										
*RT*										
No RT and RT, except IMC/SCL	185	(65.6)	189	(67.0)	ref		ref		ref	
RT to IMC/SCL	97	(34.4)	93	(33.0)	1.78	(1.13, 2.82)	1.00	(0.25, 4.01)	1.93	(1.18, 3.17)
										
*Chemotherapy*										
No	276	(97.9)	261	(92.6)	ref		ref		ref	
Yes	6	(2.1)	21	(7.4)	0.69	(0.23, 2.05)	3.58	(0.32, 40.6)	0.26	(0.06, 1.18)
										
*Tam*										
No	236	(83.7)	239	(84.8)	ref		ref		ref	
Yes	46	(16.3)	43	(15.2)	0.69	(0.37, 1.29)	0.16	(0.02, 1.35)	0.94	(0.48, 1.86)

CI=confidence interval; IMC=internal mammary chain; OR=odds ratio; RT=radiotherapy; SCL=supraclavicular; Tam=tamoxifen.

**Table 5:** Associations between laterality of BC, RT, and location of stroke

**BC-side**	**Carotis right**	**Carotis left**	***P*-value**
*Stroke*			
Right	49 (43.4)	64 (56.6)	
Left	56 (52.3)	51 (47.7)	0.22
			
RT			
Right	25 (43.9)	32 (56.1)	
Left	31 (51.7)	29 (48.3)	0.46
			
RT, except IMC/SCL			
Right	9 (45.0)	11 (55.0)	
Left	10 (50.0)	10 (50.0)	1.00
			
RT to IMC/SCL			
Right	16 (43.2)	21 (56.8)	
Left	21 (53.8)	18 (46.2)	0.37
			
			
*Ischaemic stroke and ill-defined cerebrovascular lesion*			
Right	45 (43.7)	58 (56.3)	
Left	52 (53.6)	45 (46.4)	0.20
			
RT			
Right	22 (44.9)	27 (55.1)	
Left	29 (52.7)	26 (47.3)	0.44
			
RT, except IMC/SCL			
Right	6 (37.5)	10 (62.5)	
Left	10 (50.0)	10 (50.0)	0.52
			
RT to IMC/SCL			
Right	16 (48.5)	17 (51.5)	
Left	19 (55.9)	15 (44.1)	0.63

BC=breast cancer; IMC=internal mammary chain; RT=radiotherapy; SCL=supraclavicular.

**Table 6:** ORs and 95% CIs for stroke in association with daily fraction radiation doses

	**Case**	**Control**	**OR**	**Age adjusted**
*Daily fraction radiation dose IMC*				
No RT	184	189	ref	
⩽2.5 Gy	20	34	0.56	(0.24, 1.31)
2.6–3.9 Gy	64	49	2.61	(1.48, 4.60)
⩾4 Gy	11	10	3.05	(0.97, 9.58)
Unclear	3	0	—	—
				
*Daily fraction radiation dose SCL*				
No RT	183	190	ref	
⩽2.5 Gy	19	33	0.54	(0.23, 1.29)
2.6–3.9 Gy	45	36	2.14	(1.15, 3.99)
⩾4 Gy	32	23	4.06	(1.85, 8.94)
Unclear	3	0	—	—

CI=confidence interval; IMC=internal mammary chain; OR=odds ratio; RT=radiotherapy; SCL=supraclavicular.
